# Dissociation between iron accumulation and ferritin upregulation in the aged substantia nigra: attenuation by dietary restriction

**DOI:** 10.18632/aging.101069

**Published:** 2016-10-12

**Authors:** Thomas Walker, Christos Michaelides, Antigoni Ekonomou, Kalotina Geraki, Harold G Parkes, Maria Suessmilch, Amy H Herlihy, William R Crum, Po-Wah So

**Affiliations:** ^1^ Department of Neuroimaging, Institute of Psychiatry, Psychology and Neuroscience, King's College London, London, United Kingdom; ^2^ Diamond Light Source, Harwell Science and Innovation Campus, Didcot, Oxfordshire, United Kingdom; ^3^ CR-UK Clinical MR Research Group, Institute of Cancer Research, London, United Kingdom; ^4^ Perspectum Diagnostics, Oxfordshire, United Kingdom

**Keywords:** MRI, brain, aging, iron, homeostasis, dietary restriction, basal ganglia

## Abstract

Despite regulation, brain iron increases with aging and may enhance aging processes including neuroinflammation. Increases in magnetic resonance imaging transverse relaxation rates, R2 and R2*, in the brain have been observed during aging. We show R2 and R2* correlate well with iron content via direct correlation to semi-quantitative synchrotron-based X-ray fluorescence iron mapping, with age-associated R2 and R2* increases reflecting iron accumulation. Iron accumulation was concomitant with increased ferritin immunoreactivity in basal ganglia regions except in the substantia nigra (SN). The unexpected dissociation of iron accumulation from ferritin-upregulation in the SN suggests iron dyshomeostasis in the SN. Occurring alongside microgliosis and astrogliosis, iron dyshomeotasis may contribute to the particular vulnerability of the SN. Dietary restriction (DR) has long been touted to ameliorate brain aging and we show DR attenuated agerelated in vivo R2 increases in the SN over ages 7 – 19 months, concomitant with normal iron-induction of ferritin expression and decreased microgliosis. Iron is known to induce microgliosis and conversely, microgliosis can induce iron accumulation, which of these may be the initial pathological aging event warrants further investigation. We suggest iron chelation therapies and anti-inflammatory treatments may be putative ‘antibrain aging’ therapies and combining these strategies may be synergistic.

## INTRODUCTION

Iron is essential for normal neuronal functioning [1], but in excess, labile reactive iron enhances free radical induced cell death [2]. Thus, cellular iron homeostasis is tightly regulated, including sequestration in a bioavailable and non-toxic form by ferritin, the major cellular iron storage protein [3]. However, iron does accumulate with aging (see below) and iron dysregulation (and accumulation) has been implicated in the pathogenesis of various CNS disorders including Alzheimer's Disease (AD) [4] and Parkinson's Disease (PD) [5], highlighting the key role of iron homeostasis in brain pathology.

The seminal human post-mortem study by Hallgren and Sourander (1958) showed iron accumulation within the globus pallidus (GP), striatum (STR, including both caudate and putamen), thalamus and various cortical regions [6]. Subsequent investigations have shown similar findings, with the basal ganglia exhibiting the greatest levels of iron accumulation [7]. Iron in the GP greatly increases during development, but plateaus in early adulthood, with continual iron accumulation in the STR into senescence (biological aging) [8]. Iron has also been shown to accumulate with aging in the substantia nigra (SN) [8].

The apparent iron dyshomeostasis during aging may predispose the brain to neurodegenerative disease. Indeed, the role of iron in enhancing reactive oxygen species (ROS) and mitochondrial injury is key to the “mitochondrial theory of aging” [9]. Cellular production of ROS is unavoidable with ROS substrates being by-products of normal mitochondria respiration. The brain is particularly susceptible to oxidative stress due to its high oxygen consumption; high content of peroxidizable unsaturated fatty acids; high amounts of pro-oxidants (iron and ascorbate); and poor antioxidant defenses [10]. Further, the high numbers of do-paminergic neurons in the SN are known to be continuously metabolically active and particularly prone to oxidative stress [11]. This increased metabolic burden has been suggested to accelerate the aging of SN neurons; indeed, death of dopaminergic neurons occurs at a 5-10% greater rate than any other neuronal type [12]. This innate predisposition of the SN to oxidative damage makes it particularly susceptible to deleterious iron accumulation and thus, a potential therapeutic target. Indeed, oxidative damage is very much a feature of neurodegenerative diseases and may partly explain why advancing age is a major risk factor. In *C. elegans*, aging is also associated with iron accumulation and increased dietary levels of iron were shown to weaken protein homeostasis so as to accelerate age-related protein aggregation, a feature of aging [13].

Magnetic resonance imaging (MRI) is a non-invasive modality that has been used to elucidate morphological changes with aging, demonstrating decreases in cortical and striatal brain volumes [14]. More recently, other MRI techniques including relaxometry have provided indirect evaluation of age-related changes in brain iron [15]. These methods depend on iron-induced enhancement of longitudinal (T1) and transverse (T2) relaxation of neighboring protons - predominantly affecting the latter. However, iron also induces local magnetic field inhomogeneities and increases susceptibility, shortening T2* relaxation times [16, 17]. It should be noted that the T2 measurements are also affected by diffusion of the spins during the interecho time for an applied spin-echo sequence [18]. The reciprocal of T1, T2 and T2* relaxation times are the relaxation rates, R1, R2 and R2*, respectively, thought to correlate better with iron than the former [19]. MRI has demonstrated age-related decreases of T2 and T2* (increased R2 and R2*, respectively), primarily in the basal ganglia, in both man and rhesus monkey [17, 20-23]. Higher R2 and R2* were observed in the STR, with a greater increase in the GP [22, 24], and consistent with iron accumulation in these regions (see above). Age-related alterations in relaxation times/rates have also been shown to predict age-related impaired motor function [21, 25] and cognitive dysfunction [26].

Ever since McKay et al. (1935) [27] evoked life extension in rats by restricting food intake, dietary restriction (DR) has been studied in various rodent and primate models of aging. DR is defined as reduction of a particular or total nutrient intake without causing malnutrition by reducing total food intake or inter-mittent fasting. Focused on the health-span effects of DR, i.e. time spent free of age-related illness, studies showed DR-amelioration of age-associated morbidities [28]. This is undoubtedly due to a multitude of cellular alterations stimulated by DR, such as attenuation of oxidative damage [29], including protein oxidation (33), and suppression of glycolysis [29]. DR also reduces expression of genes encoding proteins involved in the innate immune response and its oxidative capacity [30]. Attenuation of such cellular changes in aging may underlie the positive impact of DR, with cognitive and motor functions as beneficiaries of DR at the functional level [31]. The extension of health-span by DR may also be mediated by down-regulation of the mammalian molecular target of rapamycin (mTOR), since impaired mTOR has similar effects as DR, including lifespan extension [32, 33]. DR has also been known to reduce iron accumulation [34, 35], but only one study has demonstrated DR-amelioration of age-related T2 increases in the basal ganglia, combined with improved motor function [16]. Furthermore, iron chelation was shown to extend healthspan and lifespan in *C. elegans* [13]. DR also induces hormesis, acting as a mild stressor, enhancing host defense mechanisms to natural stressors encountered during life [29]. The glial activation, in the absence of neurodegeneration, observed in the aging brain, has also been shown to be attenuated by DR [36].

In this study, we tested the hypothesis that iron changes underlie the age-related alterations in MR relaxation rates in the basal ganglia and that relaxation rates provide proxy measures of iron content and/or biological brain age (study 1). R1, R2 and R2* indices in the basal ganglia of aging C57BL/6J mice were correlated with synchrotron radiation X-ray fluo-rescence (SRXRF) elemental iron maps, with ferritin immunohistochemistry performed to assess iron dyshomeostasis. Typically, R1, R2 and R2* have been correlated against previously published post-mortem, generally qualitative, histochemical Perl's staining for iron content [6]; or from bulk analysis of tissue samples [37]. By using SRXRF in our study, we obtained semi-quantitative spatial iron measures from the same brain region/tissue that relaxometry was also performed on. In addition, age-associated neuroinflammation was assessed with glial fibrillary acidic protein (GFAP) and ionized calcium binding adaptor molecule (iba1) immunohistochemistry, staining for astrocytes and microglia, respectively.

The effectiveness of MRI measure(s) to assess biological brain age was tested in a model of delayed brain aging stimulated by DR (study 2) and focused specifically in the SN, a brain area particularly vulnerable to disease [11]. DR attenuated the age-associated changes in MRI (study 1) as well as restored iron homeostasis and prevented neuroinflammation in the SN.

## RESULTS

In study 1, age-related (over 2 – 27 months) changes in relaxation rates, striatal volumes, iron, and ferritin, ba1 and GFAP IHC were investigated, whereas in study 2, we studied the effects of DR on these measurements.

### Study 1: *Ex vivo* characterization of the normally aging mouse brain

#### Age-related alterations in MR Relaxation Rates

**Figure 1:**
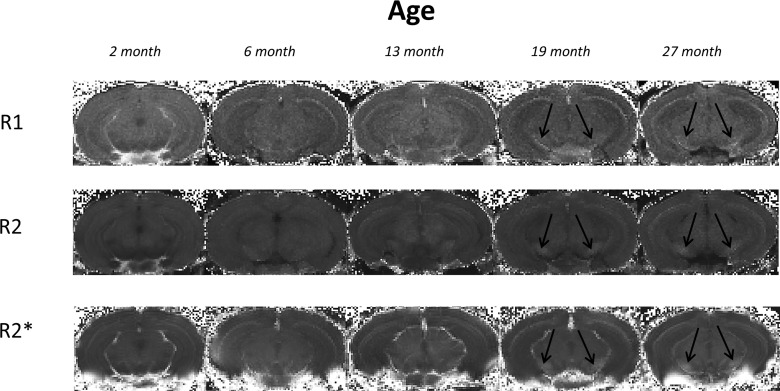
Typical *ex vivo* R1, R2 and R2* maps of brains at approximate bregma -3.28 from mice aged 2, 6, 13, 19 and 27 month in study 1. Black arrows indicate the substantia nigra.

Typical R1, R2 and R2* maps of the brain including the substantia nigra at approximate Bregma -3.28 are shown in Fig. 1.

**Figure 2:**
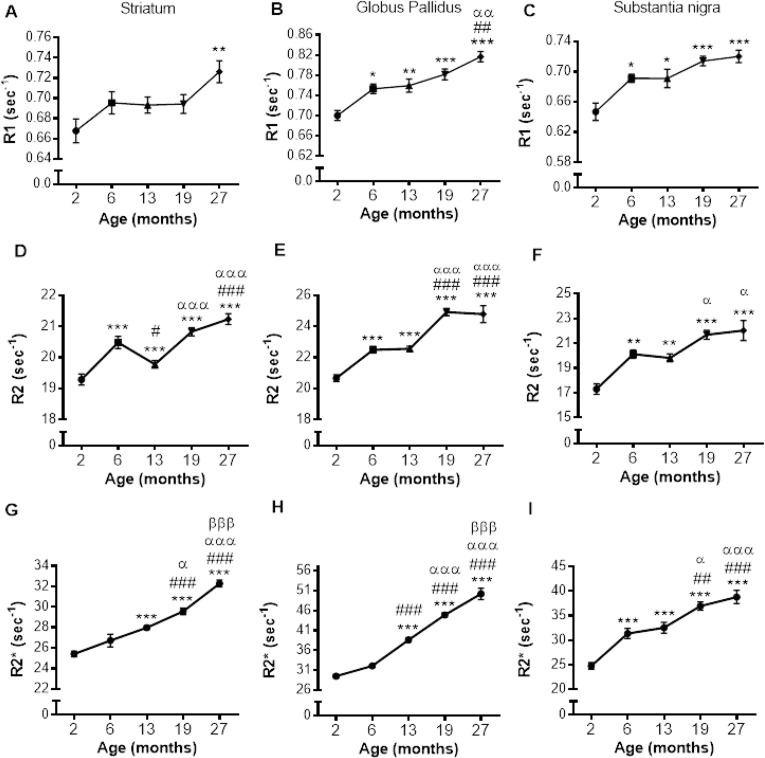
lterations in R1 (**A**-**C**), R2 (**D**-**F**) and R2* (**G**-**I**) with increasing age in the striatum, globus pallidus and substantia nigra, respectively. Significance level at *, *P* < 0.05; **, *P* < 0.01 and ***, *P* < 0.001, compared to 2 month; #, *P* < 0.05; ##, *P* < 0.01; and ###, *P* < 0.001, compared to 6 month; α, P < 0.05; αα, *P* < 0.01 and ααα, *P* < 0.001, compared to 13 month and βββ, *P* < 0.001, compared to 19 months, respectively.

**R1 relaxation rate**: R1 was significantly different in the STR, GP and SN (*P* < 0.01, < 0.0001 and < 0.0001, respectively; Fig. 2A – C; Supplemental Table 1). Post-hoc testing showed that R1 was higher in 27 than 2-month-old mice in the STR (9%; *P* < 0.01; Fig. 2A). In the GP, R1 was higher at 6, 13, 19 and 27 compared to 2 months (8, 8, 12 and 17%; *P* < 0.05, 0.01, 0.001 and 0.001, respectively). R1 was also higher in 27-month-old mice compared to those at 6 and 13 months (8%; *P* < 0.01 and 0.01, respectively; Fig. 2B). Much of the R1 increase occurred over 2 – 6 months of age in the SN with R1 higher at all ages compared to 2 month old mice (6, 13, 19 and 27 months; 7, 7, 10 and 11%; *P* < 0.05, 0.05, 0.001 and 0.001, respectively; Fig. 2C).

**R2 relaxation rate**: A pronounced effect of age on R2 was observed in the STR, GP and SN (*P* < 0.0001; Fig. 2D – F; Supplemental Table 1). In the STR, R2 was higher at 6, 19 and 27 months compared to at 2 months (6, 9 and 10%, respectively; *P* < 0.001; Fig. 2D), but a progressive increase in R2 was not observed, as R2 at 13 months was lower than at 6 months (3%; *P* < 0.05), although still higher than that at 2 months (9%; *P* < 0.001). Much of the increase in R2 was observed between 13, and 19 and 27 months with R2 being comparable at 19 and 27 months of age.

R2 was higher at 6, 13, 19 and 27 compared to 2 months in the GP (9, 9, 21 and 20%; *P* < 0.001, for all; Fig. 2E) with R2 being similar at aged 6 and 13, and 19 and 27 months. Much of the increase in R2 during aging occurred between 2 - 6 months and 13 - 19 months (9 and 10%, respectively; *P* < 0.001). The changes in R2 in the SN were similar to that observed in the GP, with R2 higher in 6, 13, 19 and 27 months compared to 2 months (16, 15, 25 and 27%; *P* < 0.01, 0.01, 0.001 and 0.001, respectively; Fig. 2F). Furthermore, much of the increase in SN R2 occurred between 2 – 6, with R2 unchanged over 6 and 13 and 19 and 27 months (Fig. 2F).

**Figure 3:**
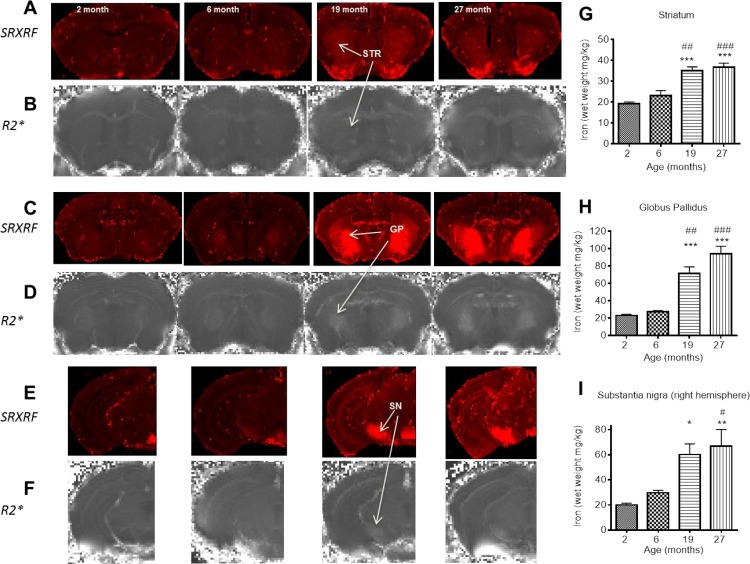
SRXRF elemental iron maps in (**A**) striatum, (**C**) globus pallidus and (**E**) substantia nigra with corresponding R2* maps in (**B**), (**D**) and (**F**) at 2, 6, 19 and 27 months. [Arrows highlight clear accumulation of iron concordant with increased R2*, most prominent between 6 and 19 months.] Qualitative increases in iron are accompanied by significant quantitative increases in iron, largely between 6 and 19 months in (**G**) striatum, (**H**) globus pallidus and I) substantia nigra. Significance level at *, *P* < 0.05; **, *P* < 0.01 and ***, *P* < 0.001, compared to 2 month; #, *P* < 0.05; ##, P < 0.01; and ###, *P* < 0.001, compared to 6 month, respectively.

R2* relaxation rate: R2* appeared to increase from 6 – 19 months in the GP and SN (Fig. 3D and F), with more modest changes in the STR (Fig. 3B). Indeed, R2* significantly differed with aging in the STR, GP and SN (*P* < 0.0001, 0.0001 and 0.0001, respectively; Supplemental Table 1).

In the STR, R2* was higher at 13, 19 and 27 compared to 2 months (10, 16 and 27%, respectively; *P* < 0.001, Fig. 2G). R2* also increased between 13 and 19 months (6%; *P* < 0.05), and 19 and 27 months (9%; *P* < 0.001). Similar R2* changes were observed in the GP, with R2* higher at 13, 19 and 27 months compared to 6 (20, 40 and 56%; *P* < 0.001) and R2* higher at 19 months compared to 13 (16%; *P* < 0.001), and 27 months higher than that at 19 (12%; *P* < 0.001, Fig. 2H).

R2* was higher at all ages compared to 2 months in the SN (6, 13, 19 and 27 months; 26, 31, 50 and 57%, respectively; *P* < 0.001; Fig. 2I). R2* at aged 13 months was lower than that at 19 and 27 months (14 and 19% *P* < 0.05 and 0.001, respectively). The significant R2* increases over 19 and 27 months observed in both the STR and GP was not observed in the SN.

#### Age-related changes in SRXRF-measured brain iron

Increased R2* clearly aligned with areas of increased iron in the SRXRF iron maps (Fig. 3A - F). Iron increased with age in the STR, GP and SN (*P* < 0.0001; Supplemental Table 1). In the STR, iron levels were similar at 2 and 6 months, but higher at 19 (82 and 52%; *P* < 0.001 and 0.01, respectively; Fig. 3G) and 27 months (91 and 59%; *P* < 0.01, respectively; Fig. 3G). The GP also had higher iron levels at 19 (210 and 160%; *P* < 0.001 and 0.001; Fig. 3H) and 27 months (307 and 242%; *P* < 0.001 and 0.001; Fig. 3H) than at 2 and 6 months, respectively. Similar findings were also observed in the SN, where levels of iron were comparable at 2 and 6 months, but higher at 19 (201%; *P* < 0.5; Fig. 3I) compared to 2 months and at 27 months (235 and 125%; *P* < 0.01 and 0.5; Fig. 3I) compared to 2 and 6 months, respectively. Whilst iron levels were similar at 19 and 27 months, only the latter was significantly different from levels at 6 months.

#### Correlation of individual relaxation rates with SRXRF measured iron

**Figure 4:**
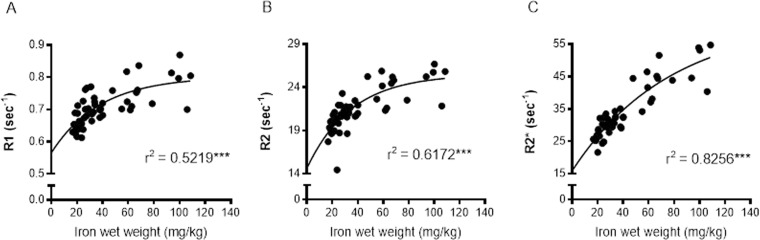
Nonlinear regression and Spearman's rank-order correlation of (**A**) R1 (**B**) R2 and (**C**) R2* with iron in the basal ganglia. A significant positive correlation of iron was found for all MR relaxation rates, but nonlinear regression demonstrated R2* to have the greatest predictive value for iron. Significance level of correlation at ***, *P* < 0.001.

Relaxation rates were correlated with SRXRF iron measurements from all three basal ganglia regions rather than individual regions to provide a wide range of iron concentrations for correlations. R1, R2 and R2* significantly positively correlated with iron content in the three basal ganglia regions, STR, GP and SN (r^2^ = 0.5219, 0.6172 and 0.8256, respectively; all correlations, *P* < 0.001; Fig. 4A-C). Nonlinear regression analysis suggests that for R1 and R2 in particular, there may be a saturation effect as at high iron levels, R1 and R2 appears to plateau. However, overall R2* correlated better with iron than R1 (*P* < 0.05) and were similar to R2.

#### Changes in striatal volume with aging

STR volumes normalized to whole brain volumes were comparable between 2 and 6 months (Supplemental Fig. 1) but lower at 13, 19 and 27 months compared to at 2 months (*P* < 0.001; Supplemental Fig. 1). STR volume at age 27 months was also lower than that at 6 month (7%, *P* < 0.05).

#### Age-related changes in ferritin-immunopositive cell numbers

**Figure 5:**
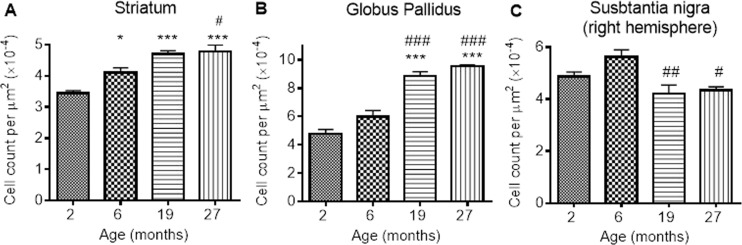
Ferritin-immunopositive cell counts increases in response to iron accumulation in the (**A**) striatum and (**B**) globus pallidus, but decreases from 6 months in (**C**) substantia nigra. Significance level at *, *P* < 0.05; and ***, *P* < 0.001, compared to 2 month; #, *P* < 0.05; ##, *P* < 0.01 and ###, *P* < 0.001, compared to 6 months, respectively.

Numbers of ferritin-immunopositive cells differed in the STR (*P* < 0.0001), GP (*P* < 0.0001) and SN (*P* < 0.01; Fig. 5A-C; Supplemental Table 1) with aging. More ferritin-immunopositive cells were observed at 6 (33%), 19 (67%) and 27 months (67%) compared to 2 months (*P* < 0.05, 0.001 and 0.001, respectively) in the STR (Fig. 5A). Ferritin-expressing cells were also elevated between 6 and 27 months (25%; *P* < 0.05), but similar at 19 and 27 months.

In the GP, numbers of ferritin-immunopositive cells were similar at 2 and 6 months, but higher at 19 and 27 months (by 80 and 100% compared to 2 months, *P* < 0.001; 50 and 67% compared to 6 months, *P* < 0.001; Fig. 5B). Numbers of ferritin-expressing cells in the SN were also similar at 2 and 6 months (Fig. 5C). However, less ferritin-immunopositive cells were detected at 19 and 27 months compared to those at 6 months (20 and 33%; *P* < 0.01 and < 0.05, respectively).

#### Neuroinflammation during aging

**Figure 6:**
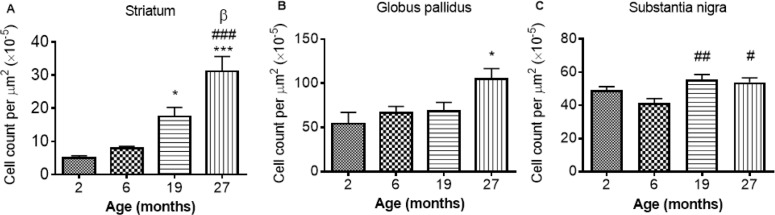
GFAP cell count per μm2 increases with age in the (**A**) striatum (**B**) globus pallidus and (**C**) substantia nigra. Significance level at *, *P* < 0.05 and ***, *P* < 0.001, compared to 2 month; #, *P* < 0.05; ##, *P* < 0.01; and ###, *P* < 0.001, compared to 6 month and β, *P* < 0.05, compared to 19 months, respectively.

**GFAP-immunohistochemistry**: The number of GFAP-positive cells was significantly different between the ages in the STR, GP and SN (*P* < 0.0001, < 0.01, < 0.05, respectively; Fig. 6A-C; Supplemental Table 1). Similar levels of GFAP cells were observed between 2 and 6 months in the STR, with 27 month mice exhibiting higher numbers of astrocytes than 2 (529%), 6 (292%) and 19 months old (78%) (compared with 2 and 6 months, *P* < 0.001; compared with 19 months *P* < 0.05). 19 month mice also expressed higher levels of GFAP than 2 month-old mice (253%; *P* < 0.05) (Fig. 6A). Little change was observed in GFAP-positive cells in the GP between 2, 6 and 19 months, but 27 month mice had higher levels than 2 month-old mice (94%; *P* < 0.05).

In the SN, comparable levels of GFAP were observed from 2 - 6 and 19 - 27 months, with 19 and 27 months expressing higher levels of GFAP than 6 months (35 and 30%, *P* < 0.01 and 0.05, respectively; Fig. 6C).

**Figure 7:**
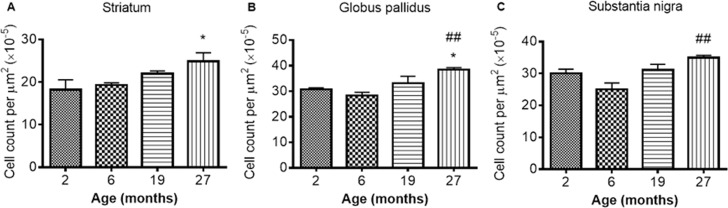
Iba1 cell count increases with age in the (**A**) striatum (**B**) globus pallidus and (**C**) substantia nigra at different ages. Significance level at *, *P* < 0.05, compared to 2 month and ##, *P* < 0.01, compared to 6 month, respectively.

**Iba1 immunohistochemistry**: Iba1 immunopositive cell counts were significantly altered with aging in the STR, SN and GP (*P* < 0.05, 0.01 and 0.01 respectively; Fig 7A-C; Supplemental Table 1). Post-hoc testing showed that Iba1 cells were significantly higher at 27 months compared to 2 months in both the STR (Fig. 7A) and GP (Fig. 7B) (*P* < 0.05; 36 and 25% respective-ly). In the SN (Fig. 7C) and GP (Fig. 7B), Iba1 positive cells were higher at 27 months than at 6 months (*P* < 0.01, 39 and 36% respectively). Iba1 positive cells were unchanged between 2 and 6 and 19 - 27 months in all 3 studied basal ganglia regions (Fig. 7A-C).

### Study 2: Dietary restriction (DR)-induced attenuation of age-related brain changes in the SN

#### Effect of DR on in vivo R2 and ferritin immunoreactivity

**Figure 8:**
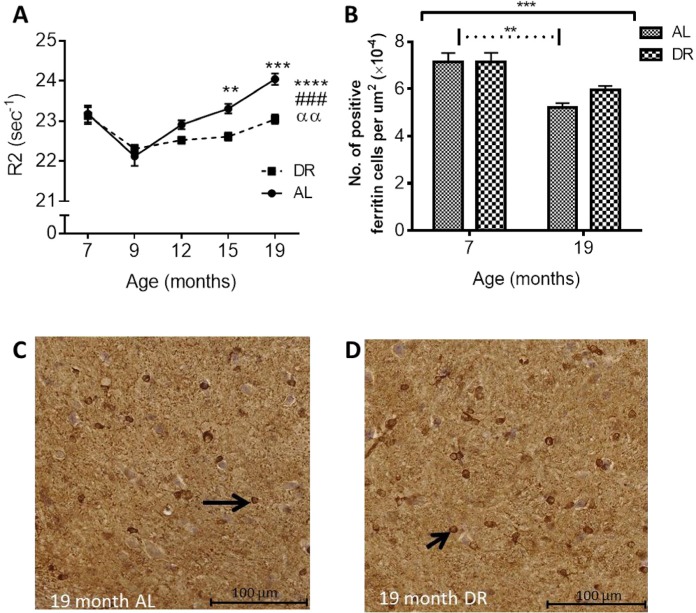
(**A**) Dietary restriction ameliorates age-related increases in *in vivo* R2 in the SN. Black asterisks placed above specific age time points indicate differences between Ad libitum (AL) and dietary restricted (DR) mice after post-hoc test (significance levels ** *P* < 0.01 and *** *P* < 0.001). Significance level at **** *P* < 0.0001 for an effect of age when placed at the end of the graph; ### *P* < 0.001 for an effect of treatment; αα, *P* < 0.01 for an age-feeding regimen interaction. (**B**) Age-related reductions in ferritin-immunopositive cell numbers in the SN between 7 and 19 months are ameliorated in DR mice compared to AL mice. [Solid black line indicates the outcome of two-way ANOVA testing; *** *P* < 0.001 for effect of age; no significant effect of DR or age-feeding regimen interaction. Dotted line indicates post-hoc testing at significance level ** *P* < 0.01.] Micrographs showing decreased ferritin-positive cells in SN of (**C**) AL compared to (**D**) DR mice at 19 months of age. Black arrows point to ferritin-immunopositive cells.

In vivo R2: Aging led to an increase in *in vivo* R2 in the SN, similar to that observed in study 1 (*P* < 0.0001; Fig. 8A). A significant effect of DR (*P* < 0.001) was also observed, as well as an interaction between age and feeding regimen (*P* < 0.01). DR and *ad libitum* (AL) mice both showed similar decreases in R2 at ages 7 and 9 months, with little change to 12 months (Fig. 8A). After 12 months of age, R2 in AL mice increases with little change in DR mice (Fig. 8A). Multiple comparison testing showed progressive disparity between AL and DR groups at aged 15 and 19 months (3 and 4%, respec-tively; *P* < 0.01 and *P* < 0.001, respectively; Fig. 8A).

**Ferritin immunohistochemistry**: Ferritin-immuno-positive cell numbers in the SN decreased with aging as in study 1 (*P* < 0.001) and multiple comparisons testing showed the reduction in cell numbers was only significant in the AL and not DR mice (27% and 18%, respectively; *P* < 0.01; Fig. 8B-D). An effect of feeding regimen or an interaction between feeding regimen and age was not observed.

#### Effect of dietary restriction on neuroinflammation

**Figure 9:**
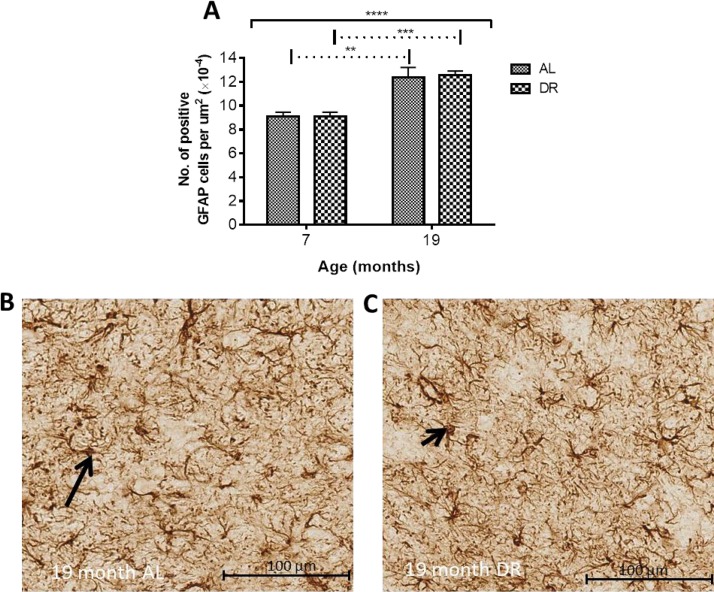
(**A**) Dietary restriction has no effect on age-related increases in GFAP positive astrocytes. Solid black line indicates the outcome of two-way ANOVA testing; **** *P* < 0.0001 for effect of age; no significant effect of DR or age-feeding regimen interaction. Dotted line indicates post-hoc testing at the significance levels ** *P* < 0.01 and *** *P* < 0.001. Micrographs showing no difference in GFAP positive astrocytes between (**B**) AL and (**C**) DR mice at 19 months. Black arrows point to GFAP-immunopositive cells.

**GFAP Immunohistochemistry**: Numbers of GFAP immunopositive cells were significantly altered with age in the SN (*P* < 0.001). No effect of DR or an interaction between DR and age was observed in the SN. Multiple comparisons testing revealed numbers of GFAP-positive cells were increased in both AL and DR mice at 19 compared to 7 months (36% and 38%, *P* < 0.01 and 0.001; Fig. 9A-C).

**Figure 10:**
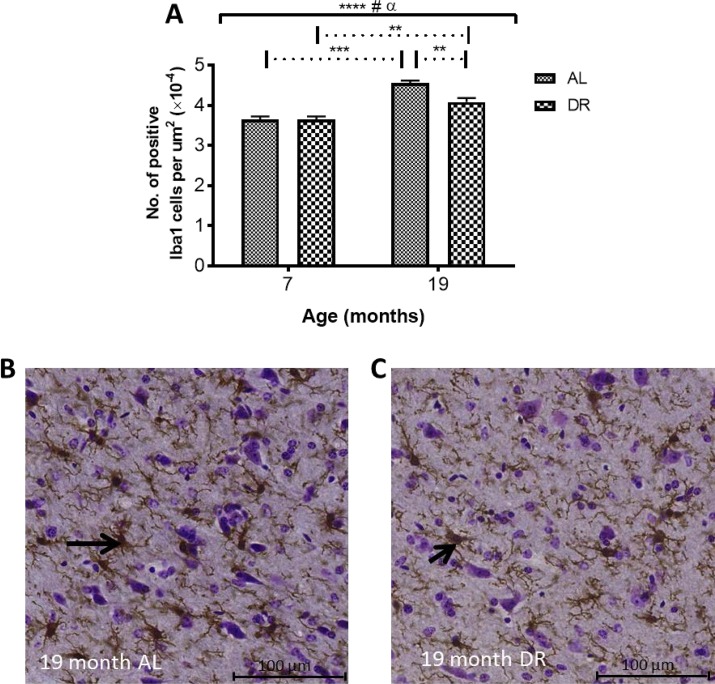
(**A**) Dietary restriction reduces age-related increases in Iba1 in DR compared to AL mice. Solid black line indicates the outcome of two-way ANOVA testing; **** *P* < 0.0001 for effect of age; # *P* < 0.05 for an effect of treatment; α, *P* < 0.05 for an age-feeding regimen interaction. Dotted line indicates post-hoc testing at the significance levels ** *P* < 0.01 and *** *P* < 0.001.] Micrographs showing higher numbers of Iba1-positive cells in (**B**) AL compared to (**C**) DR mice at 19 months. Black arrows point to Iba1-immunopositive cells.

**Iba-1 immunohistochemistry**: Two-way ANOVA showed a significant effect of age in the SN (*P* < 0.0001). There was also a significant effect of DR (*P* < 0.05) and an interaction between age and DR (*P* < 0.05). Iba1 cell numbers were elevated in both AL (25%, *P* < 0.001) and DR mice (12%, *P* < 0.01; Fig. 10A) with post-hoc multiple comparisons tests revealing significantly lower numbers of Iba1 positive cells in DR compared to AL mice at 19 months (10%, *P* < 0.01; Fig. 10A–C).

## DISCUSSION

This is the first study to show DR-induced amelioration of iron dyshomeostasis in the SN, a region known to be particularly vulnerable to disease, intimating a potential pivotal role for SN in development of the aging phenotype. Our results highlight iron chelation therapies as potential putative “anti-brain aging” interventions, which may also attenuate the risk of developing neurodegenerative diseases, particularly Parkinson's disease.

In the STR, we observed a continual increase in R2* with aging, consistent with human studies [22, 38]. We show increased R2* was correlated with increased iron, at least up to 19 months of age. Maximum iron content was observed at 19 months in mice, consistent with similar observations in man [6] at corresponding ages [39]. Whilst human studies showed significant correlations of R2* to histochemical-detected iron [6], we show R2* correlations with direct semi-quantitative iron measurements, as well as within the same spatial region of the same sample.

In the GP, rapid increases in R2* [22] and iron [6] during early life have been shown in humans, followed by a plateau at 30 - 40 years of age. No initial rapid increase in R2* or iron in the GP was observed in our study. However, Haacke et al., (2010) [38] did report similar findings to those reported here in humans of a comparable age range to mice in our study, i.e., with the youngest being young adults as opposed to children as in the Aquino et al., (2009) study [22]. Whilst full correspondence to human investigations cannot necessarily be expected, these results intimate a need for developmental aging to be distinguished from senescence, at least when considering changes in iron and relaxation rates.

Our finding of increased R2* in the STR and GP between 19 - 27 months without concomitant increased accumulation of iron is noteworthy, although in the latter, there was a trend towards increased iron albeit significance was not reached. The increased R2* in the STR, alongside unchanged levels of iron, suggest iron is partitioning into different forms with aging. Iron is often associated with ferritin but may also bind to hemosiderin and magnetite. Hemosiderin-iron is an aggregate that induces greater spin dephasing [40] and so has a greater effect on T2* (and thus R2*) than ferritin-iron, which acts mostly through T2 relaxation [40]. Iron associated with biogenic magnetite also has a higher magnetic moment than that stored in ferritin and better at enhancing T2 relaxation [41]. A shift in the proportion of iron to these more magnetically active forms in the STR could thus account for R2* increases, without concomitant increases in iron. One study has reported increased hemosiderin in the aged (>65 years of age) putamen, a sub-region of the STR [42].

Aquino et al., (2009) showed age-related increases in SN R2* over the lifespan (1 – 80 years) [22], similar to that reported here. However, one post-mortem study showed the majority of iron accumulation occurred very early in life [43] and another, a more linear increase [44]. The apparently contradictory findings between these two human studies most likely arise from differences in sample age ranges, again highlighting the need to distinguish developmental from biological aging. The trajectory of iron accumulation in our study is consistent with that of Zecca et al., (2004) [44], who used a similar sample age range.

Both R1 and R2 values in the basal ganglia exhibited an overall increase with age. Whilst both measures significantly correlated to iron as in human studies [45], we showed a reduced correlation between R1 and iron as previously reported [46, 47]. Interestingly, for R1 and R2 particular, nonlinear regression analysis showed these relaxation times tended to plateau at higher iron concentrations, suggesting there is a saturation point for the influence of iron on these relaxation rates: this saturation effect was not readily observed for R2*. It was noted R2 remained a reliable predictor for changes in brain iron during senescence, with a greater influence of other tissue factors on R2 occurring up to 13 months of age (see below).

R2 values were similar between 6 - 13 months in the GP and SN, with a significant decrease observed in the STR, despite iron accumulation, which would increase R2. Astrogliosis has been shown to increase T2 (decrease R2) [48] potentially by enlarging the extracellular space and increasing interstitial fluid content [49-51]. Indeed, astrocytes are more reactive with aging [52] and the demonstrable increased astrogliosis shown in the STR here likely accounts for decreases in R2 observed between 6 and 13 months, without a measurable decrease in STR volume. The decreased R2 in the STR may also result from neuronal loss, although unlikely, given the lack of atrophy over 6 – 19 months. Alterations in cellular content and tissue structure clearly play a prevalent role on age-related change in R2, at least up to 13 months. This may potentially mask the influence of iron accumulation on R2 to this point and may also explain why studies with narrower age ranges have reported no age-related alterations in T2 [53, 54]. Of note, after 13 months, R2 changes in all the basal ganglia regions mirrors R2*, demonstrating R2 is as suitable a proxy for iron content as R2* in advanced aging.

Overall, the smallest effect of age was seen on R1 relaxation rate, which we have shown to have the least, albeit significant, correlation to iron. The overall pattern of R1 change across the mouse lifespan reflects the increase in iron, but without the specificity of R2* or even R2.

DR has previously been demonstrated as a robust “anti-brain aging” intervention [27]. We have highlighted the SN as a potentially crucial region of deleterious iron accumulation in aging, especially due to its high neuronal vulnerability [11]. In this context, our most pertinent finding is that DR ameliorates *in vivo* age-related R2 increases in the SN alongside inhibited iron-induced up-regulation of ferritin. Other studies have also shown DR-amelioration of increases in T2 with aging [16], but not in the serial manner here or with corroborating ferritin IHC measurements. The demons-trable strength of *ex vivo* R2 to predict elevations in iron (particularly during senescence) in the current investigation lends credence that the amelioration of age-related increases in *in vivo* R2 following 12 months pertain to DR-attenuation of iron accumulation.

Under normal homeostatic control, increased cellular iron induces expression of ferritin to increase iron-storage capacity [55]. Indeed, increased iron in the GP throughout aging, alongside increased ferritin-positive cells shown here, has been documented previously [56]. In the STR, higher ferritin-immunopositive cell numbers preceded iron accumulation over 2 – 6 months, but did not continue to increase over 6 – 19 months, despite increased iron, suggesting that ferritin is only partially loaded with iron in the STR of younger brains. Ferritin can store up to 4500 atoms of iron and in peripheral tissues at least, ferritin remains only partially saturated with iron [57]. Increased ferritin-immuno-positive cell numbers at 27 months compared to 6 suggest that existing ferritin levels are insufficient to accommodate the increasing iron levels. Studies in rodents and man have also shown increased ferritin in the STR with aging [55, 56], with one rat study showing a similar pattern of iron and ferritin change in the STR as we have reported here [55].

Unexpectedly, the increased iron levels in the SN over 6 and 19 months were accompanied by a decrease, rather than an increase, in ferritin-immunopositive cells, suggesting iron dysregulation. The impaired iron handling/storage with aging in the SN may predispose this region to oxidative damage and subsequent neurodegeneration, and contribute to the select neuronal vulnerability of this region [11]. Rather than being sequestered in ferritin, the excess iron may be sequestered in neuromelanin (NM), given the prevalence of NM-containing dopaminergic neurons in the SN [58]. Indeed, NM is known to associate with iron and shown to increase with aging [43, 44]. Furthermore, Zecca et al., (2004) reported impaired ferritin expression in aged SN neurons in man alongside significantly large amounts of NM-bound iron. NM may protect neurons against toxic quinone and semi-quinone, by-products of dopamine metabolism [59], with the SN having the highest dopaminergic neuronal content. However, at high iron concentrations, NM becomes saturated, leading to a potentially pro-oxidant environment [60, 61] and indeed high levels of NM, have been associated with SN neurodegeneration in middle-aged mice [62]. Whether excess iron is indeed sequestered by NM in aging and if so, whether the iron level reported here is sufficient to saturate NM in the SN, warrant future investigation.

Iron mishandling in the SN may have prevalent effects throughout the brain as SN dopaminergic projections modulate striatal neurons [63], affecting sensorimotor learning and memory [64]. Further, decreases in nigral neurons with aging [65] may precipitate impaired dopaminergic innervation and consequent behavioral abnormalities [66]. Indeed, studies have shown D_1_ and D_2_ dopamine receptor losses in the STR with aging [67], as well as a reduced ligand binding [68]. Others have shown that the caudal and lateral STR, receiving afferent input from the A9 SN neurons, exhibit decreases in dopamine content [69]. In light of this, iron dyshomeostasis in the SN demonstrated here, may play a prevalent and crucial role in stimulating oxidative damage of the melanized dopaminergic neurons, contributing to their vulnerability and explaining the extrapyramidal symptoms of the aging brain.

In PD, death of dopaminergic neurons in the SN pars compacta decreases dopamine levels in the prefrontal cortex and STR [70], leading to cognitive and motor dysfunction [71]. A feature of PD is the greater iron accumulation in the SN than in aging [5, 72] and similar to the current investigation, iron-induced ferritin expression is also inhibited [73]. Additionally, in-creased redox activity in NM aggregates of melanized neurons has been observed in PD, with NM-positive staining shown to positively correlate with ferric iron content [61]. The potential role of NM in PD pathophysiology poses an interesting question regarding its role in iron sequestration with respect to normal aging, given the similarity in iron alterations of our study to that reported previously in PD [73].

We have shown age-related increases in GFAP-positive astrocytes in the basal ganglia regions, consistent with previous studies [74, 75]. The expanded end feet of astrocytes encompass the brain capillary endothelial cells (BCECs) [76] and facilitate the transport of various metabolites and metals, including iron, into the neuropil [76]. Astrocytes not only take up iron as transferrin-bound iron, they can also import iron via the divalent metal transporter 1 (DMT1), which are expressed at astrocytic endfeet [77]. We show that the time-course of the increases in GFAP-immunopositive cells during aging was similar to that for iron accumulation in the STR and the SN, and may arise from the known uptake and storage of iron in astrocytes [75]. Indeed, one third to three quarters of the iron in the brain is stored in glial cells [78]. The increases in iron in the STR, possibly from increased astrocytic uptake and storage, is associated with a corresponding increase in ferritin-immunopositive cells, suggesting the increased iron is stored with ferritin. However, in the SN, the increases in both astrocytes and iron levels are not concomitant with an increase in ferritin-positive cells. As mentioned before, the excess iron does not appear to be bound by ferritin and may become dependent on NM for non-toxic storage.

In the GP, however, astrocyte levels were comparable except between 2 and 27 months, despite increased iron during this time. Iron can still be accumulating in astrocytes in the GP, without a corresponding increase in astrocytes numbers, as the astrocytes numbers in the GP at 2 months are relatively high compared to those in the STR, and appear to be sufficient to accommodate the increase in iron.

A pro-inflammatory state has been demonstrated in aging, with increases in glial cell number [79] and an activated, dystrophic microglial phenotype previously reported. Stereotactic injections of lipopolysaccharide in the STR have been shown to increase microglial activation, ferritin expression and total nigral iron content in aged rats [80], with activated microglia shown to increase iron uptake and decrease export [81]. Thus, the microgliosis observed with aging in the three basal ganglia regions may, at least in part, explain the iron accumulation in these areas. Further, microgliosis in the STR was less pronounced and correlated with lower levels of iron accumulation. Increased microglial iron uptake may induce pro-inflammatory cytokine release and neurodegeneration [82, 83]. Activated microglia produce superoxide, which is capable of stimulating iron release from ferritin [84], leading to increased labile iron, enhancing pro-oxidative capacity that is central to neuroinflammatory and neuro-degenerative processes in aging. Indeed, Hayashi et al., (2008), highlighted microglia as a major source of oxidation products in the aged mouse brain [85].

The decreased ferritin storage potential of iron in the SN reported in the current investigation alongside astrogliosis, combined with the tendency for aged microglia to become dystrophic and render iron more redox-active [84, 86], cements the SN as a particularly vulnerable region in aging. The interplay between iron accumulation and glial cell populations requires further exploration to elucidate the full extent to which this region contributes to the aged phenotype.

Whether microgliosis precedes and induces the increases in iron uptake and/or whether iron accumulation occurs initially and contributes to microglial activation/dystrophy remains to be seen. In regard to the latter, the role of microglia in long term storage of iron has previously been noted, with storage of iron potentially increasing microglial dystrophy [86]. Reduced iron accumulation, as suggested by T2, has been associated with improved motor performance [16]. Iron may have a critical role in the age-related increase and activation of microglia in aging. In light of this, the demonstrable reduction of microglia in the SN by DR, combined with apparent amelioration of iron dyshomeostasis, highlights that restoration of iron homeostasis could be a key facet of the beneficial effects of DR [35]. Determining if a causal relationship exists between the apparent restoration of iron homeostasis and reduced microglia, or if the reverse is true, could help prove the efficacy of iron chelation therapies or anti-inflammatory treatments, respectively. However, combining the two therapeutic strategies may prove to be synergistic. Indeed, interactions of inflammatory signaling molecules and iron in PD have been discussed in Pretorius et al., (2014) [87] and suggested to enhance eryptosis in the periphery. Whether the effects of DR in the SN shown here can incite beneficial changes in other regions known to be affected during aging, such as the STR that receives significant dopaminergic innervation from the SN, remains to be seen.

In addition, the lack of an astrocytic reaction, concomitant with iron dyshomestasis and microgliosis certainly supports the idea that microglia rather than astrocytes, plays a more dominant role in the handling of iron in the SN. The lack of effect of DR on astrocytes in the SN is interesting given previous reports of DR reducing age-related increases GFAP, albeit not specifically in the SN [88].

The integration of *in vivo* and *in vitro* iron measures alongside ferritin and glial immunohistochemistry in the aged mouse brain provides formative insight into the regulation of brain iron in aging, but also the importance of iron dyshomeostasis and neuro-inflammation on brain aging and as possible targets for novel interventions. Further, we reveal the particular vulnerability of the SN to iron dyshomeostasis in aging, potentially mediating/resulting from age-related inflammation and contributing to the susceptibility of this brain area to neurodegenerative diseases, especially PD. In this context, DR, and in the future DR analogues, may serve as putative interventions against such deleterious aging processes.

## METHODS

### Ethical statement

All experimental procedures performed on mice were in compliance with the local ethical review panel of King's College London and the U.K. Home Office Animals Scientific Procedures Act 1986.

### Animals and Treatment

#### Study 1*: Ex vivo* characterization of the aging mouse brain

Male C57BL/6J mouse brains were obtained from Shared Aging Research Models, (ShARM, Sheffield, UK). Mice were culled at 2 (n = 11), 6 (n = 8), 13 (n = 11), 19 (n = 11) and 27 months (n = 8) of age by rising CO_2_ inhalation (different numbers of mice were included at each age, depending on tissue availability.). Ages of mice chosen for study were equivalent to different stages of adulthood from just post-adolescence, adult, midlife, elderly and the very elderly. The heads were removed and fixed in 4% paraformal-dehyde (1 week) and stored in phosphate-buffered saline (PBS, 4°C) with 0.05% sodium azide prior to MRI, SRXRF and immunohistochemistry (see below).

#### Study 2: DR-induced attenuation of age-related brain changes

Animals were maintained under standard laboratory conditions (room temperature automatically maintained at 21°C ± 1°C; 12h light:dark cycle). Mouse cages were provided with wood shavings and shredded paper bedding for enrichment. Body weights and food intake data were recorded weekly.

Male C57BL/6J mice (n = 24, aged 7 months old; Harlan, UK), were singly housed and acclimatized for 7 days before allocation into two groups of 12. Following *in vivo* baseline MRI (see below), one group was fed *ad libitum* (AL), while the other, underwent an intermittent fasting regimen involving fasting on alternate days from 17:30 – 11:00 h. Mice were scanned again *in vivo* at 9, 12, 15 and 19 months of age (see section on *in vivo* MRI below). After MRI at 19 months, mice underwent transcardial perfusion with heparinized (50 IU/ml) saline to flush out blood from the vasculature before perfusion-fixation with 4% paraformaldehyde under terminal anaesthesia. Heads were decapitated and fixed for a further week in 4% paraformaldehyde before storage in 0.05 % azide-supplemented PBS at 4°C until *ex vivo* MRI (see below).

### MR acquisition

***Ex vivo* MRI**: Brains within the skull were prepared for MRI (see below) by removal of, the skin, muscle, lower jaw, tongue, nasal bones and zygomatic arches, and embedded in perfluoropolyether (Galden™, Performance Fluids, UK, Solvoy Solexis) to minimize susceptibility and prevent tissue dehydration during scanning. Care was taken to avoid air bubbles. MRI was performed using a quadrature volume radiofrequency coil (33 mm internal diameter; Rapid Biomedical, Rimpar, Germany) on a 7T horizontal bore MRI scanner (Agilent Technologies Inc, Walnut Creek, CA, USA).

T1 relaxometry was achieved with a 2D spin-echo (SE) sequence with repetition times (TR) of 670, 800, 1000, 1200, 2000 and 4000 ms; echo time (TE), 11 ms and 2 averages. A 2D multi-echo, SE sequence was run with TE, 11.6, 34.8, 58.0, 81.2, 104.4, 127.6, 150.8 and 174.0 ms; TR, 7600 ms and 4 averages, to measure T2. To account for stimulated echoes only data from the odd TEs were included in the T2 fitting (see below). T2* was assessed with a 2D multi-echo gradient echo sequence with five echoes and TE, 2.5, 8.5, 14.5, 20.5, 26.5 ms; TR, 2000 ms and 10 averages, flip angle 90°. Contiguous coronal slices (40, 0.5 mm thick) were recorded with field of view (FOV), 30 × 30 mm and matrix size, 256 × 256 for all relaxometry.

***In vivo* MRI**: T2 relaxometry was performed as for brain samples at baseline (7 months), 9, 12, 15 and 19 months but with TE (10.6, 21.3, 31.9, 42.6, 53.2, 63.9, 74.5, 85.2, 95.8, 106.5, 117.1, 127.8, 138.4, 149.1, 159.7 and 170.4 ms) and TR, 5217 ms; 2 averages and 30 contiguous coronal slices at 0.5 mm thick; FOV, 20 × 20 mm and matrix size = 128 × 128.

### MRI image analysis

T1, T2 and T2* maps were generated by pixel-by-pixel non-linear fitting to equations: y = A (1-exp^(−TR/T1)^), y = B(exp^(−TE/T2)^) and y = C(exp^(−TE/T2*)^), respectively (A, B and C are constants that represents signal components that were unchanged in each experiment) in JIM 5.0 (Xinapse Systems, Alwincle, UK). R1, R2 and R2* maps (R2 only for study 2) were then calculated by performing the reciprocal function for each maps. Structural images were produced by summing the shortest 8 echoes of the T2 relaxometry data using proprietary scanner software and these images used for manual tracing of the striatum for volumetric assessment.

Regions of interest (ROIs) were manually drawn in the STR, GP and SN on the high quality structural images (guidelines listed in supplemental Table 2, according to a standard mouse brain atlas [89]) using ImageJ (National Institute of Health, Maryland, USA), and then placed onto each of the directly registered relaxation rate maps. Note the structural images were produced from the T2 relaxometry data (see above). As all relaxometry data was collected in the same scan session without moving the brains, accurate registration between the structural image and all relaxometry maps was possible. Relaxation rates for each anatomical ROI were obtained and averaged for each age before statistical testing in Prism v5 (GraphPad, SD, USA). For volume analysis, striatal ROIs were delineated as previously described [90].

### Cryoprotection and tissue sectioning

Following MRI acquisition, brains from 5 mice per group from both studies (not including 13 month old mice from study 1 as these brains were obtained sometime after synchrotron access) were cryoprotected in 30% sucrose (PBS, 0.05% sodium azide). Subsequently, cryosectioning was performed coronally to produce 40 μm thick frozen sections that were mounted onto 4 μm thick Ultralene film (Spex Sample-Prep, NJ, USA) secured to a customized holder for SRXRF. Cryosections sections of 20 μm thick were also obtained and mounted onto Superfrost plus microscope slides for IHC. Not all the brains at the various ages that had previously undergone MRI were analyzed by SRXRF as synchrotron access is very limited. Likewise, only sufficient numbers of brains at the various ages needed for statistical testing were analyzed by IHC.

### SRXRF elemental mapping and analysis

SRXRF of whole/right hemisphere of brain tissue sections was performed on the I18 beamline at the Diamond Light Source synchrotron radiation facility (Didcot, UK). The beam energy was tuned to 11 keV and focused to 100 × 100 μm (determining resolution) and the brains scanned in a raster manner. The brain samples were mounted at a 45° angle with respect to the incoming X-ray beam and the detector to minimize scatter contribution. The raw data consist of full energy dispersive spectra for each sample point exposed to the beam. The spectra were subsequently fitted and the net peak areas of the characteristic K_α_ and K_β_ peaks of iron were evaluated using PyMca [91]. Quantification was performed by measuring a reference metal film (AXO, Dresden, GmbH), which allows estimation of the photon flux on the samples. SRXRF elemental maps of pixel-by-pixel iron concentrations (mg/kg wet weight) were manually aligned to the corresponding relaxation rate maps using ImageJ. Thus, ROIs, similar to that for the relaxation maps, could be placed at the equivalent spatial locations on the elemental iron maps to quantify regional iron.

### Immunohistochemistry

Standard DAB IHC was performed. Endogenous peroxidase activity was blocked by incubation with 1% H_2_O_2_ in Tris- buffered saline, pH 7.4 containing 0.2% Triton X-100 (TBS+; 30 mins, RT). After 2 × 5 mins washes in TBS+, non-specific binding was blocked using 10% skimmed milk power (SMP) in TBS+ (2 h, RT). Sections were then incubated with either rabbit anti-ferritin antibody (F6136; Sigma-Aldrich, Poole, Dorset, UK), rabbit polyclonal anti-Iba-1 antibody (019-19741; Wako Pure Chemical Industries, Richmond, VA), rabbit polyclonal anti-GFAP antibody (DAKO, High Wycombe, UK) in 5% SMP in TBS+ (overnight, 4°C). After washes in TBS+, slides were incubated with secondary biotinylated anti-rabbit IgG (1:200, DAKO, High Wycombe, UK) in 5% SMP in TBS+ (2 h, RT). Avidin-biotin binding was then performed using the ELITE ABC kit (Vector, Peterborough, UK) for ferritin and Iba1 immunohistochemical protocols, respectively, prior to the chromogenic reaction using the Impact DAB peroxidase kit (Vector, Peterborough, UK). Slides were subsequently dehydrated in ascending grades of ethanol, cleared in xylene and mounted using DPX (Sigma-Aldrich, St Louis, MO, USA).

### Histological image analysis

Sections were scanned on a LEICA SCN400F scanner (University College London, London) to produce 20× magnification digital images from 4 sections per mouse in the STR (Bregma +1.10 to 0.50 mm), GP (Bregma -0.22 to -0.70mm) and SN (Bregma -3.08 to -3.64 mm) from the right hemisphere for each section. Depending on the region, 2 – 4 optical fields were taken, each comprising a 628.0 × 278.5 μm^2^ area. Ferritin, GFAP or iba1 immunopositive cells in each optical field were manually counted using the cell counter plugin of ImageJ and expressed as number of immunopositive cells per unit area.

### Data analysis

Spearman's rank-order correlation and nonlinear regression analysis was performed to assess the strength of the association between R1, R2 and R2* with SRXRF measured elemental iron in all basal ganglia regions. Statistical testing of correlations was performed using cocor [92].

One way analysis of variance (ANOVA) with post-hoc Tukey's correction (Prism v5, GraphPad, San Diego, CA, USA) was used to test for differences in individual relaxation rates, iron, ferritin, Iba1 and GFAP-immunopositive cells and normalized STR volume with aging (study 1). A two-way ANOVA with repeated measures and post-hoc Sidak's multiple comparisons test was used for testing differences in *in vivo* R2 with aging and feeding regimen (study 2). Two-way ANOVA was also used to test for differences in ferritin, Iba1 and GFAP-immunopositive cell numbers between AL and DR mice in study 2.

All values quoted are mean ± standard error of mean (SEM) and significance was set at *P* < 0.05.

## SUPPLEMENTARY MATERIAL TABLES AND FIGURE


