# Supplementary material for: Dissociation between iron accumulation and ferritin upregulation in the aged substantia nigra: attenuation by dietary restriction
Source: Aging (Albany NY). 2016 Oct 12;8(10):2488–506. doi: 10.18632/aging.101069 (PMC5115902; doi:10.18632/aging.101069)
Supplement: Supplementary file 1 [file aging-08-2488-s001.pdf]

SUPPLEMENTARY MATERIAL

Supplemental Table 1. R1, R2 and R2* relaxation rates; synchrotron radiation-based X-ray fluorescence measured iron; ferritin, GFAP and iba1 immunopositive cell counts in the striatum, globus pallidus and substantia nigra; and striatal volumes in C57Bl/6 mice aged 2, 6, 13, 19 and 27 months. Values are mean ± SEM values.

Measurement	Brain region	Age				
		2 month	6 month	13 month	19 month	27 month
R1 relaxation rate	Striatum	0.67 ± 0.01	0.70 ± 0.01	0.69 ± 0.02	0.69 ± 0.01	0.73 ± 0.01
	Globus Pallidus	0.70 ± 0.01	0.75 ± 0.01	0.76 ± 0.01	0.78 ± 0.01	0.82 ± 0.01
	Substantia Nigra	0.65 ± 0.01	0.69 ± 0.01	0.69 ± 0.01	0.71 ± 0.01	0.72 ± 0.01
R2 relaxation rate	Striatum	19.3 ± 0.18	20.5 ± 0.20	19.8 ± 0.12	20.8 ± 0.13	21.2 ± 0.17
	Globus Pallidus	20.7 ± 0.22	22.5 ± 0.21	22.6 ± 0.16	24.9 ± 0.23	24.8 ± 0.55
	Substantia Nigra	17.3 ± 0.42	20.1 ± 0.33	19.8 ± 0.33	21.7 ± 0.37	22.0 ± 0.80
R2 relaxation rate*	Striatum	25.4 ± 0.28	26.7 ± 0.62	28.0 ± 0.18	29.6 ± 0.32	32.3 ± 0.96
	Globus Pallidus	29.5 ± 0.43	32.1 ± 0.34	38.6 ± 0.53	44.8 ± 0.52	50.2 ± 1.41
	Substantia Nigra	24.7 ± 0.67	31.4 ± 1.01	32.5 ± 1.10	37.0 ± 0.82	38.8 ± 1.34
Synchrotron radiation-based X-ray Fluorescence measured iron (wet weight mg/kg)	Striatum	19.3 ± 0.78	23.2 ± 2.31	Not measured	35.2 ± 1.76	36.9 ± 1.85
	Globus Pallidus	23.1 ± 1.31	27.5 ± 1.28	Not measured	71.5 ± 7.63	94.1 ± 8.86
	Substantia Nigra	20.0 ± 1.44	29.7 ± 1.96	Not measured	60.2 ± 8.43	66.9 ± 13.2
Ferritin (10^{-4} cell counts/μm^2)	Striatum	3.4 ± 0.1	4.1 ± 0.1	Not measured	5.8 ± 0.2	5.9 ± 0.2
	Globus Pallidus	4.8 ± 0.3	6.0 ± 0.4	Not measured	8.8 ± 0.3	9.5 ± 0.1
	Substantia Nigra	4.9 ± 0.2	5.6 ± 0.3	Not measured	4.2 ± 0.3	4.3 ± 0.1
GFAP (10^{-5} cell counts/μm^2)	Striatum	5.0 ± 0.7	8.0 ± 0.6	Not measured	17.5 ± 0.3	31.2 ± 0.5
	Globus Pallidus	54.0 ± 13.0	66.8 ± 6.91	Not measured	68.1 ± 10.3	104.7 ± 12.0
	Substantia Nigra	49.3 ± 2.0	41.3 ± 2.8	Not measured	55.6 ± 3.0	53.8 ± 2.8
Iba1 (10^{-5}cell counts/μm^2)	Striatum	18.2 ± 2.2	19.3 ± 0.5	Not measured	22.0 ± 0.6	24.9 ± 2.0
	Globus Pallidus	30. 7 ± 0.6	28.3 ± 1.3	Not measured	33.1 ± 2.7	38.5 ± 0.7
	Substantia Nigra	30.0 ± 1.3	25.1 ± 1.9	Not measured	31.1 ± 1.7	35.0 ± 0.7
Normalized volume (mm^3)	Striatum	0.0429 ± 0.0005	0.0406 ± 0.0006	0.0387 ± 0.0010	0.0383 ± 0.0003	0.0376 ± 0.0008

Supplemental Table 2. Guidelines for delineation of basal ganglia ROIs for MRI and SR-XRF data analysis.

Brain region	Approximate bregma (mm)	Description
**Striatum**	+0.62	Elliptical ROI placed under the corpus callosum close to, but ensuring not in the lateral ventricles
**Globus Pallidus**	-0.34/-0.46	Elliptical ROI, with the right edge lined up with the lateral ventricles and left edge delineated by the contrast difference between the striatum and globus pallidus. Height defined by the space between the 3rd dorsal ventricle and the 3rd ventricle.
**Substantia Nigra**	-3.28	Small elliptical ROI placed alongside the visible cerebral peduncle at the base of the ventral hippocampus.

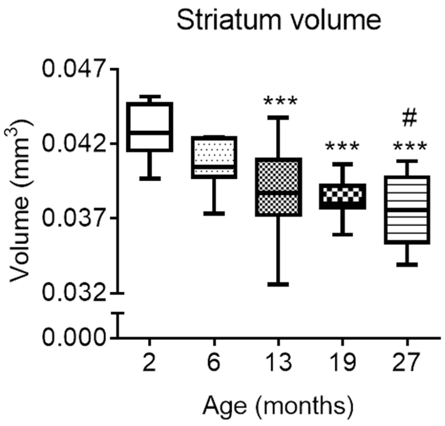

Supplemental Figure 1. Normalized striatal volumes decrease steadily with advancing age. Significance level at ***, $P < 0.001$, compared to 2 month and #, $P < 0.05$ compared to 6 months, respectively.
